# Novel Weapons Testing: Are Invasive Plants More Chemically Defended than Native Plants?

**DOI:** 10.1371/journal.pone.0010429

**Published:** 2010-05-03

**Authors:** Eric M. Lind, John D. Parker

**Affiliations:** Smithsonian Environmental Research Center, Edgewater, Maryland, United States of America; CNRS UMR 8079/Université Paris-Sud, France

## Abstract

**Background:**

Exotic species have been hypothesized to successfully invade new habitats by virtue of possessing novel biochemistry that repels native enemies. Despite the pivotal long-term consequences of invasion for native food-webs, to date there are no experimental studies examining directly whether exotic plants are any more or less biochemically deterrent than native plants to native herbivores.

**Methodology/Principal Findings:**

In a direct test of this hypothesis using herbivore feeding assays with chemical extracts from 19 invasive plants and 21 co-occurring native plants, we show that invasive plant biochemistry is no more deterrent (on average) to a native generalist herbivore than extracts from native plants. There was no relationship between extract deterrence and length of time since introduction, suggesting that time has not mitigated putative biochemical novelty. Moreover, the least deterrent plant extracts were from the most abundant species in the field, a pattern that held for both native and exotic plants. Analysis of chemical deterrence in context with morphological defenses and growth-related traits showed that native and exotic plants had similar trade-offs among traits.

**Conclusions/Significance:**

Overall, our results suggest that particular invasive species may possess deterrent secondary chemistry, but it does not appear to be a general pattern resulting from evolutionary mismatches between exotic plants and native herbivores. Thus, fundamentally similar processes may promote the ecological success of both native and exotic species.

## Introduction

The tremendous ecological and economic costs of biological invasions have prompted intense interest in the mechanisms that control invasion success [1], [2]. The widely-cited enemy release hypothesis posits that invaders succeed by escaping their coevolved natural enemies from their home range and avoiding accrual of new enemies in their introduced range [3], [4], [5]. Invasive species are often attacked by fewer species of insects, parasites, and pathogens in their new ranges [6], [7], and a biochemical explanation has often been invoked to explain these patterns. The novel weapons hypothesis, for example, proposes that exotic species gain a competitive advantage in their new range because native enemies are not adapted to detoxifying their novel biochemistry [8], [9]. In support of this hypothesis, Cappuccino and Arnason [10] showed that invasive plants possess anti-herbivore chemistry that is generally unique from compounds in the native flora, implying that exotic plants are more chemically defended than native plants.

Paradoxically, however, the evolutionary novelty argument can also be used to support the opposite conclusion. Anti-herbivore defenses may be evolutionarily novel but *ineffective* given that they evolved to repel enemies and competitors in the old but not the new range, whereas native plants may in fact be better defended than exotics because they have long experienced natural selection from their co-occurring native enemies [5], [11]. In support of this hypothesis, native herbivores can preferentially attack exotic over native plants [12], and suppress the abundance of exotics in field experiments [13]. Evolutionary novelty has thus been argued to both suppress and promote plant invasions. However, despite the long-term negative consequences for invasions of unpalatable plants into native food-webs, to date there are no experimental studies examining whether exotic plant biochemistry is any more or less deterrent than native plants to native herbivores.

Here, we conducted a direct comparison of the deterrence value of chemistry extracted from 19 highly invasive introduced plants and 21 co-occurring native species, using bioassays with a native generalist insect herbivore to test whether exotic and thus relatively novel biochemistry has anti-feedant properties. We also examined whether chemical deterrence to herbivory was mitigated by time elapsed in the new range in the putative absence of enemies. To further determine whether chemical deterrence was related to ecological success, we examined whether abundant plants had more chemically deterrent extracts than less abundant species. Finally, to gain a broader understanding of plant defense strategies, we quantified and determined trade-offs among leaf traits related to growth and defense (leaf toughness, trichome density, specific leaf area, and percent water, C, N, P, and protein).

## Results

Relatedness according to a community phylogeny of the plant species under study explained little variance in most measured traits. Only the density of leaf trichomes showed a significant phylogenetic signal, though it was generally weak and a poor fit (λ = 0.60, P_χ2_ = 0.15). All other traits showed a fit of λ close to zero with goodness-of-fit P<0.05, indicating poor fit to a model of phylogenetic influence on trait values (Table S2).

**Figure 1:**
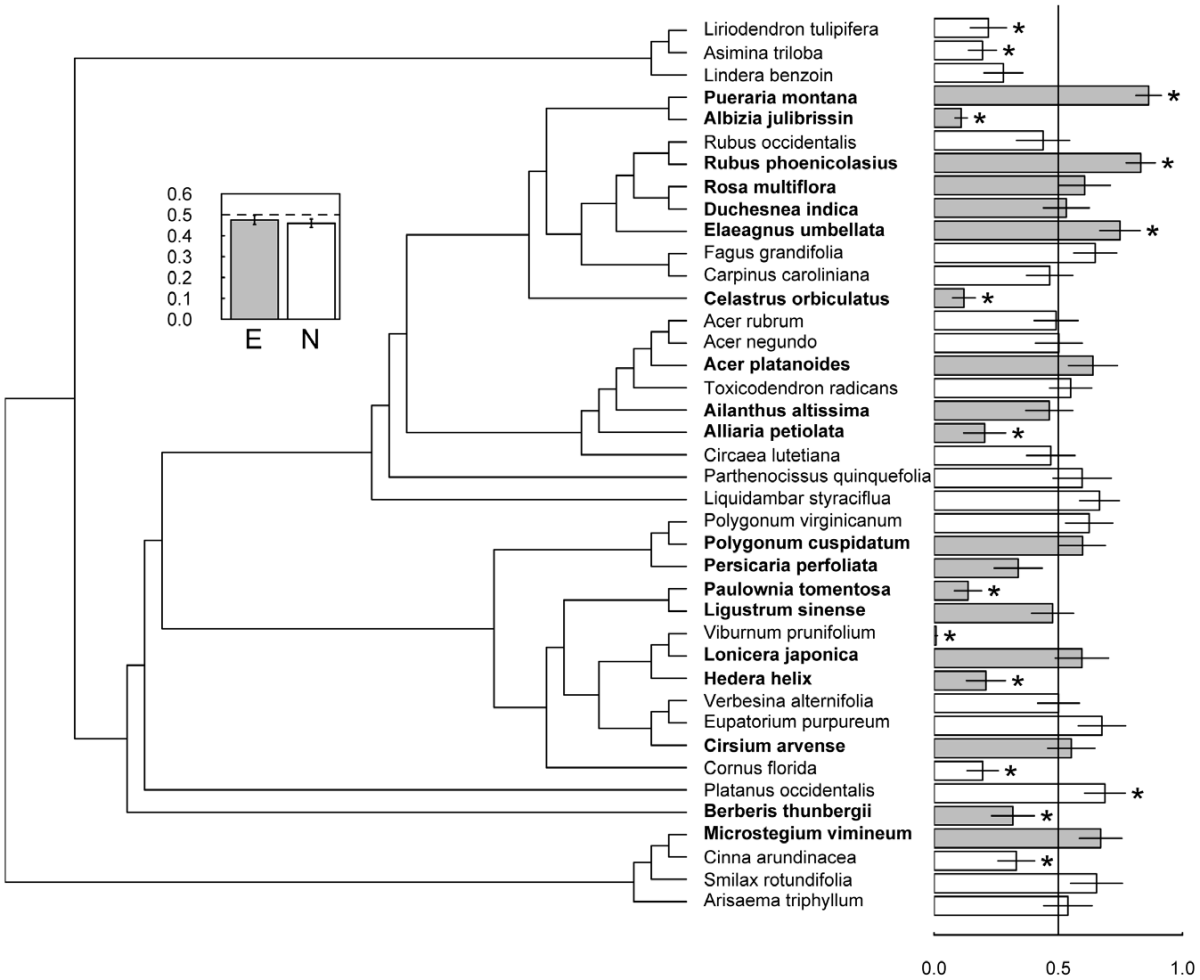
Plant origin does not predict native caterpillar preference for extracted chemistry. Preference of caterpillars for secondary chemistry extracted from 21 native (open bars) and 19 exotic (filled bars) plant species. Bars represent mean (±1 SE) fraction of extract eaten relative to total amount of diet eaten. Phylogenetic relationships are shown without branch lengths for clarity, although branch lengths were used in statistical analysis of phylogenetic influence (see text). Asterisks beside bars denote significantly (P<0.05, paired t-tests) deterrent or stimulatory extracts from individual plant species. Inset: overall mean (±1 SE) fraction eaten by species origin (ANCOVA with caterpillar mass as covariate; origin effect P = 0.76).

There was no effect of plant origin (native versus exotic) on woolly bear feeding preference for plant secondary chemistry (Fig 1 inset; mixed model ANCOVA F_1,38_ = 0.2186, P = 0.76). Caterpillar mass did influence the amount of total feeding and was therefore kept as a covariate in the analysis (ANCOVA F_1,38_ = 4.43, P = 0.02). Caterpillars had distinct preferences among extracts from different plant species (Fig 1), including 15 that were significantly stimulatory or deterrent. Extract from native sycamore (*Platanus occidentalis* L.) was significantly stimulatory (Fig. 1), whereas strongly deterrent chemistry was found in five native species, including viburnum (*Viburnum prunifolium* L.), tulip poplar (*Liriodendron tulipifera* L.), paw paw (*Asimina triloba* L. [Dunal]), flowering dogwood (*Cornus florida* L.), and the grass sweet woodreed (*Cinna arundinaceae* L.). Among exotics, significantly deterrent chemistry was found in six species, including albizia (*Albizia julibrissin* Durazz.), Chinese bittersweet (*Celastrus orbiculatus* Thunb.), garlic mustard (*Alliaria petiolata* (M. Bieb.) Cavara & Grande), princess tree (*Paulownia tomentosa* (Thunb.) Siebold & Zucc. ex Steud.), English ivy (*Hedera helix* L.), and barberry (*Berberis thunbergii* DC., Fig. 1). In contrast, caterpillars significantly preferred secondary chemistry extracted from such highly invasive exotics as kudzu (*Pueraria montana* (Lour.) Merr.), wineberry (*Rubus phoenicolasius* Maxim.), and autumn olive (*Elaeagnus umbellata* Thunb).

**Figure 2:**
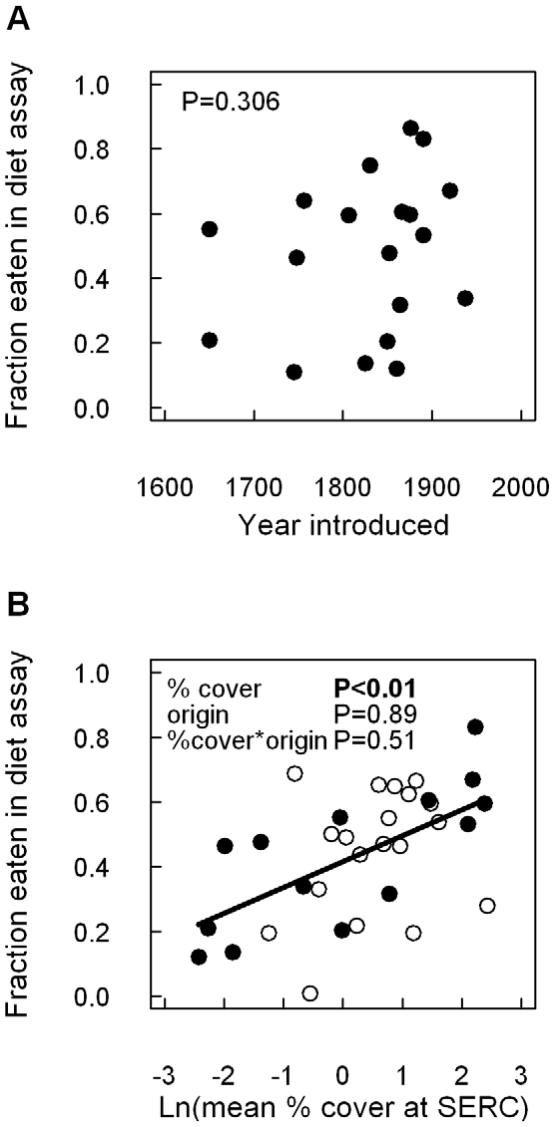
Local abundance, but not time since introduction, predicts native herbivore preference for extracted exotic plant chemistry. Relationship between leaf extract consumption and (A) date of introduction and (B) local plant abundance. There was no relationship between the earliest recorded date of introduction and extract deterrence to a native herbivore (A). However, plant species with deterrent secondary chemistry were the least abundant species in forest understory communities (B), a pattern that held across both native and exotic species (non-significant origin*abundance interaction). Filled circles represent exotic species; open circles represent native species.

There was no relationship between the earliest recorded date of invasion and extract deterrence in either linear or quadratic regression models, suggesting that time since invasion has not mitigated biochemical deterrence (data shown in Fig 2a; linear regression model F_1,17_ = 0.42, P = 0.31; quadratic regression model F_2,16_ = 0.55, P = 0.59). The most abundant species in forest understory communities at SERC had the most palatable biochemistry (Fig 2b; F_1,29_ = 12.69, P = 0.002),a pattern that held across both native and exotic species (non-significant origin*abundance interaction, F_1,29_ = 0.4382, P = 0.513, Fig. 2b). No leaf quality traits were predictive of extract palatability (P>0.1, data not shown).

**Figure 3:**
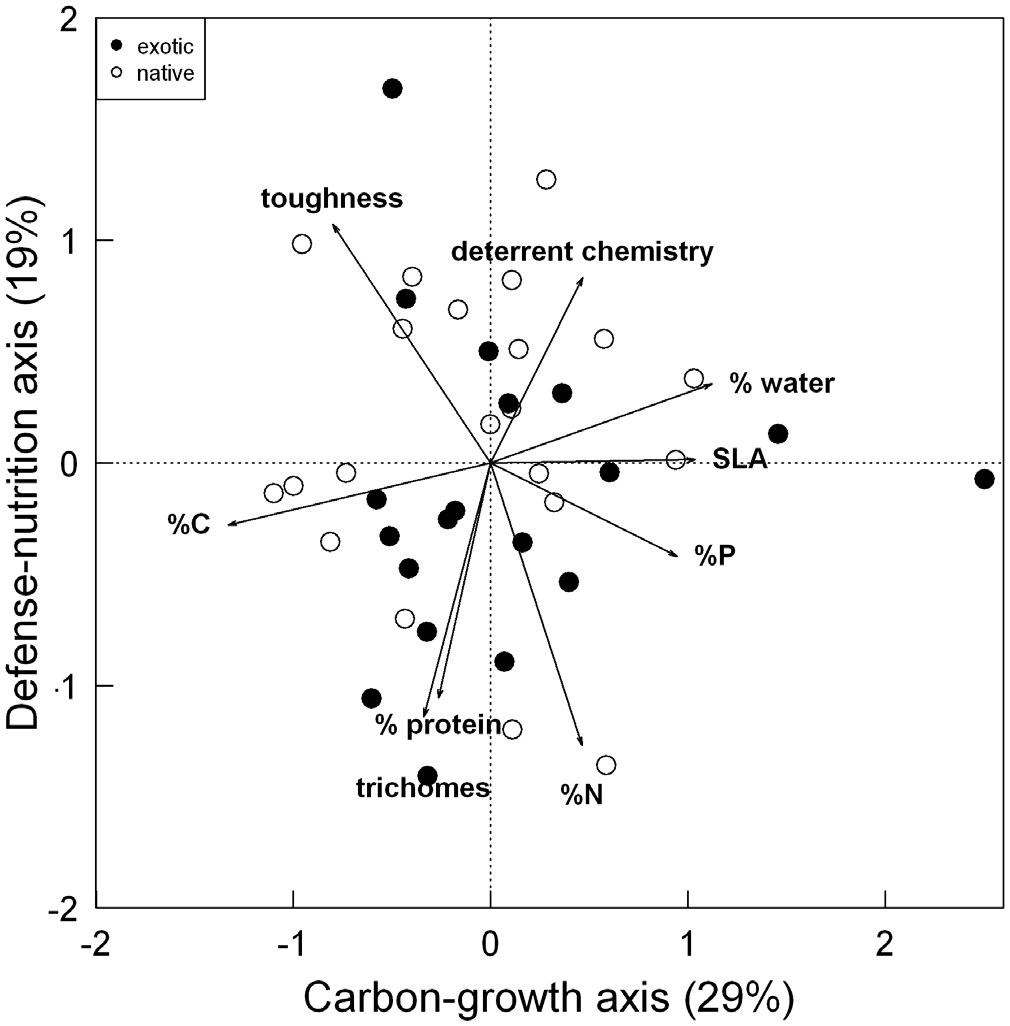
Native and exotic plant species occupy similar leaf trait space. Principal components biplot of native (open circles) and exotic (filled circles) species and their measured traits. PCA axis 1 (carbon-growth) explains 29% of trait variation across 19 exotic and 21 native species; PCA axis 2 (defense-nutrition) explains 19% of the remaining variation. All trait vectors are significantly associated with species PCA position according to randomization tests. Units for traits are given in Table 1.

Overall, the general suite of traits that we measured did not differ among native versus exotic plants (Table 1; MANOVA F_8,38_ = 0.787, P = 0.618), although exotics had significantly higher %P content on average than did native species (MANOVA F_1,38_ = 4.61, P = 0.04). Similarly, PCA analysis showed no multivariate trait differences among native versus exotic plants (all models of PCA axes ∼ origin P>0.05), but did reveal significant correlation structure among leaf traits across species (pairwise correlations in Table S3). The primary axis of variation, for example, separated species based on differential investment into structure and growth: some species had relatively carbon-rich, tough leaves whereas others had higher SLA, water, and %P content (Fig 3; axis loadings in Table S4). The secondary, orthogonal axis of variation separated species by high levels of soluble protein (and corresponding high %N) and high densities of leaf trichomes versus species that had glabrous, tough, and chemically defended leaves (Fig 3).

**Table 1:** Mean (± SE) values for nine measured traits of 19 exotic and 21 native species co-occurring in forest understory communities.

Trait	Native	Exotic	MANOVA F_1,38_
% Water‡	72.08±1.92	72.1±2.02	0.001
SLA (g cm^−2^)	369.8±34.87	358.24±36.66	0.052
Toughness (N)	133.73±9.77	131.62±10.27	0.022
Trichomes (cm^−2^)†	93.39±1016.3	1765.89±1068.4	0.634
% C‡	45.55±0.52	44.85±0.55	0.848
% N‡	2.49±0.16	2.75±0.17	1.115
% P‡	0.23±0.03	0.31±0.03	**4.608** *
% Soluble protein‡	11.81±0.85	13.25±0.89	1.560
Fraction eaten	0.46±0.05	0.47±0.05	0.025

Traits %water, SLA, toughness, trichomes are calculated from N = 20 individual plant replicates per species; %C, %N, %P, and % soluble protein calculated from N = 5 replicates per species; fraction eaten calculated from N = 20 caterpillar bioassays per species.

Overall MANOVA F_9,30_ = 0.842, p = 0.584.

† =  log-transformed for analysis.

‡ =  arcsin-square root transformed for analysis.

* =  P<0.05.

## Discussion

By virtue of being evolutionarily novel, the secondary chemistry of exotic plants has been hypothesized to be both more and less bioactive than native plants [5], [8], [9], [10], [13]. Overall, our results with direct feeding assays on plant secondary chemistry isolated from leaf tissues do not support either hypothesis. Extracts from our 19 exotic species, all of which are considered invasive, were no more chemically deterrent than extracts from 21 co-occurring native species (Fig. 1). Instead, the native woolly bear caterpillar (*P. isabella)* readily consumed secondary chemistry from nine exotic species, strongly avoided chemistry from six exotics, and preferred the secondary chemistry of three others (Fig 1). Patterns were similar for native species. Moreover, there was no relationship between extract deterrence and the amount of time since introduction (Fig. 2a), suggesting that bioactivity has not simply been lost over time in response to enemy release [e.g.], [ 14,15]. These results suggest that although particular invasive species may possess deterrent secondary chemistry, it does not appear to be a general pattern resulting from evolutionary mismatches between exotic plants and native herbivores.

We utilized direct comparisons of artificial diets that differed solely in plant chemistry to test the hypothesis that novel biochemistry is deterrent to naïve herbivores. This methodology has been widely used to directly test the effectiveness of chemical defenses in isolation from other plant traits [16], [17], and when used in conjunction with quantification of these additional traits, it can give a robust and nuanced picture of plant defense syndromes in response to herbivory [e.g.], [ 18], [19,20]. Although preference for plant leaf chemistry in isolation might not reflect herbivore feeding preferences in nature, particularly if extrinsic factors like predation and parasitism shift herbivore feeding to non-preferred host-plants [21], [22], [23], extracts of species known to be utilized by woolly bears in the field were either readily consumed or preferred (e.g., *Acer*, *Eupatorium*, *Parthenocissus quinquefolia* (L.) Planch., *Platanus occidentalis*), and caterpillars similarly avoided extracts from native plants known to be chemically defended against generalist Lepidoptera, including paw paw [24], tulip poplar [25], and flowering dogwood [26]. Thus, although other herbivores might respond differently to the same chemistry, our laboratory assays isolating plant chemistry alone reflected known woolly bear feeding preferences in nature.

Understanding the outcome of evolutionarily novel plant-herbivore interactions requires a mechanistic understanding of how herbivores perceive and detoxify novel secondary metabolites, and how plants perceive and respond to feeding by novel herbivores [27]. We focused on total feeding response by the herbivore and not the mechanisms generating these responses. However, our study had five species and one congener of a species overlapping with Cappuccino and Arnason [10], a study that did document the identity of a plant's primary defensive compounds, their relative uniqueness to compounds found in the native flora, and thus their potential to act as novel weapons. Of these six species, four had secondary compounds that were rare or absent in the native flora and thus were considered potentially deterrent. In our study, two of these (*Alliaria petiolata* and *Celastrus orbiculatus*) were deterrent, one (*Polygonum cuspidatum*) was readily consumed, and one (*Elaeagnus umbellata* Thunb.) was stimulatory (Fig 1). Of the two species identified as having relatively common and thus potentially non-deterrent secondary chemistry, one species (*Berberis thunbergii*) was deterrent and the other (*Pueraria montana*) was stimulatory (Fig 1). These disparate results emphasize that interactions between herbivores and particular defensive compounds can result in both positive and negative feeding responses regardless of chemical novelty [27], and that characterization of plant chemical defenses should rely on direct rather than indirect tests.

A key question in invasion biology has been whether exotic and native species differ along the worldwide leaf economics spectrum, and specifically whether exotics tend to possess traits related to rapid-growth and low-defense [e.g., high SLA, short leaf lifespan, few defenses; 28]. In general, successful invaders do appear shifted along the leaf economics spectrum towards a faster growth strategy than non-invaders, often possessing higher relative growth rates due to enhanced photosynthetic capacity, greater SLA, and increased foliar N and P [29], [30], [31], [32], [33], [34], [35], [36], [37], [38], . Consistent with these studies, we found that exotics had higher %P in leaf tissues relative to natives (Table 1). P content can be indicative of rapid cell turnover and high growth rates [40], [41], suggesting that the exotics in our study may gain a competitive advantage over natives by growing more rapidly.

In contrast, we did not find differences among natives and exotics in %N or SLA (Table 1), nor did we find differences when we compared traits in a multivariate context (Fig 3). One hypothesis for the general lack of differences among natives and exotics in our study is that overall environmental conditions at our study site are selecting for species with largely similar suites of traits [e.g.], [ 39,42]. For example, the 40 species in our study are characteristic of early and mid-successional secondary forests recovering from intensive agricultural use [43], and these habitats are dominated by intensive herbivory by white-tailed deer *Odocoileus virginianus* (personal observations). Thus, one hypothesis is that deer herbivory coupled with successional dynamics may favor both native and exotic species with traits promoting rapid growth and tolerance of herbivory rather than chemical deterrence [44].

The roles of host plant quality and secondary chemistry on herbivore feeding preferences have been extensively studied [45], but relatively little is known about the influence of local plant community composition on innate preferences [46]. A surprising finding in our study is that caterpillar feeding on chemical extracts was positively related to abundance of plant species in the field (Fig 3), especially given that host-plant quality is generally expected to trump the effects of host abundance on feeding preferences [46]. However, caterpillars used in our feeding trials were reared in the lab from eggs on a nutritionally complete diet and fed nutritionally equivalent test foods that differed solely in the presence of plant extracts. Thus, feeding rates should reflect innate preferences rather than nutritional differences or inadequacies [47]. Moreover, we found no relationships between extract palatability and plant nutritional quality (%N, %P, or % protein), suggesting that plant chemistry was not an indirect cue for plant quality. Interestingly, some Arctiid moth caterpillars are considered ‘toxic plant generalists’ that readily feed on chemically noxious plants to self-medicate against parasitism [23]. Thus, Arctiids like the one we studied might be predisposed to feeding on chemically-rich plants, although it is unclear if preferential ingestion of toxic foods still occurs in the absence of parasitoids (as was the case for our laboratory-reared population). Thus, we speculate that our findings could indicate an adaptive response to experience with locally abundant hosts, particularly for mobile generalists (like *P. isabella*) that individually feed on multiple plants throughout their lifetime. To our knowledge, this would be the first example that plant chemistry alone can be an imprinted cue indicative of local host-plant abundance irrespective of plant quality.

Exotic plants have been described as being both well-defended and highly competitive [e.g., 48], implying they are disconnected from the normal growth-defense tradeoffs faced by their native counterparts. However, we examined 40 co-occurring species and found that both native and exotic plants existed in fundamentally similar multivariate trait space (Fig 3). This indicates that native and exotic plants experience similar ecophysiological constraints. Moreover, the abundances of both native and exotic plants in field settings were negatively related to chemical deterrence (Fig 2b), suggesting that neither group in this system is succeeding by being chemically deterrent to herbivores. These broadly convergent patterns support the idea that similar processes can promote both native and exotic species [49], and that biochemical novelty does not necessarily lead to invasion success.

## Materials and Methods

### Study site and species

We conducted our study in natural areas in and around the Smithsonian Environmental Research Center (SERC) located along the western shore of the Chesapeake Bay in Edgewater, MD (38°53′ N, 76°33′ W). SERC and the surrounding areas are semi-rural and comprised of old fields, croplands, and forests. Nearly all forests are secondary, post-agricultural forests within which biological invasions are common [43], [50], [51]. We focused on 40 understory plant species that are abundant at SERC and in surrounding areas [43, Parker et al. unpublished data], 21 species native to eastern Maryland and 19 introduced species, the majority of Eurasian origin (Figure 1, Table S1). All 19 exotic species in our study have been categorized as invasive to the mid-Atlantic region of USA (http://www.invasive.org/maweeds.cfm accessed December 2009). Plant material was collected on or immediately adjacent to SERC property in mid- to late summer spanning two growing seasons (2008–2009, Table S1). For each plant species, we collected 10 fully expanded, undamaged leaves from 20 individuals or patches (in the case of grasses and clonal species). From each individual plant or clonal patch, one leaf was randomly selected and measured for specific leaf area (SLA), toughness, trichome density, and percent water (Text S1). Half of the remaining leaf material was dried, ground, and analyzed for %C, %N, %P and % soluble protein (Text S1); the remaining leaves were frozen at −20°C.

### Caterpillar preference assays

To prepare extracts, previously frozen leaf tissues were coarsely ground, added to a beaker, and extracted with a series of hydrophilic to lipophilic solvents (1∶1 v/v of water∶methanol, 2∶1 v/v of methanol∶dichloromethane, and 2∶1 v/v dichloromethane∶methanol). Combined extracts were condensed by evaporating the solvents under vacuum and then incorporated into an artificial diet presented in choice tests against a control diet to a common generalist herbivore, the woolly bear caterpillar (*Pyrrharctia isabella* J.E. Smith; details in Text S1). Woolly bears are ubiquitous herbivores in the forest understory and feed on a wide variety of plants including many with deterrent secondary chemistry [52]; this allowed us to systematically quantify the chemical deterrence of multiple plant species against a common enemy [e.g.], [ 18,19]. Caterpillars were presented with two weighed diet portions, one test portion containing plant extracts and a control portion lacking extracts but otherwise treated identically. Preference was quantified as mass eaten corrected for expected mass loss due to diet evaporation over the course of each individual feeding trial (Text S1).

### Statistical analyses

Phylogenetic relatedness among the studied plant community can result in convergent suites of plant traits that similarly influence herbivore preference [53]. To account for the influence of shared evolutionary history on our measured plant traits, we used phylocom software [54] to estimate a community phylogeny based on a compiled angiosperm phylogeny (P.F. Stevens, Angiosperm Phylogeny website http://www.mobot.org/MOBOT/research/APweb/ accessed November 2009). We further resolved polytomies within the phylogeny using other published phylogenetic hypotheses ([55] for the genus *Acer*, [56] for the family Rosaeae). For each of the measured traits we estimated phylogenetic signal by fitting Pagel's λ using maximum likelihood, and used chi-square goodness of fit tests to compare the fit value to λ = 1, representing phylogenetic determination of traits [57], [58]. Traits with fit that were not significantly different from λ = 1 were analyzed with generalized least squares (GLS) using the phylogeny to estimate covariance among trait values across species based on Brownian evolution [59]. Traits not showing significant phylogenetic signal were analyzed using linear mixed effects models not corrected for phylogenetic relatedness, but with species as a random effect nested in plant origin (native or exotic). Analyses were conducted in R v2.10 [60] using the ape, geiger, and nlme packages.

We tested overall preference of caterpillars (mass test diet eaten - mass control diet eaten) for native versus exotic plant extracts with mass of the assay caterpillar as a covariate to control for the total amount of feeding in each replicate assay. For each individual plant species, we used a paired t-test to examine whether extracts had significantly deterrent or stimulatory properties (test versus control diet eaten in N = 20 assays per species).

For exotic species, we used linear and quadratic regression models to test the hypothesis that extract palatability was related to time since invasion. For both native and exotic plant species, we used linear regression models to test the hypotheses that extract palatability was related to abundance (factorial with species origin), and plant leaf quality (%N, %P, %protein; each factorial with species origin). We used multiple analysis of variance (MANOVA) to examine the effects of species origin on plant traits while controlling for covariance among traits. The species mean value of each of trait was used and transformed where necessary to meet assumptions of linearity. Finally, we examined correlations among leaf traits in combination using principle components analysis (PCA). The resulting orthogonal axes of variation were then tested independently for differences among native versus exotic plants (e.g., PCA axis 1 ∼ origin) using linear models. All statistical analyses were performed using R software v2.10 [60].

## Supporting Information

Text S1 Detailed methods for data collection and analysis.
(0.10 MB DOC)

Table S1 Taxonomic, origin, and trait data for species used in the study. Data are species means based on the indicated replications per species. Diet preference represents the mean fraction consumed of artificial diet with extracts from each species versus a neutral control diet (0 =  all control preferred, 1 =  all species extract preferred). Origin and date introduced for exotic species compiled from www.invasive.org. “NA”  =  North America (native species).
(0.10 MB DOC)

Table S2 Estimates of Pagel's λ showing influence of assembled phylogeny on trait variance across species. For each trait individually, species mean values and the assembled phylogeny (see supplemental methods) were used to fit a λ value with restricted maximum likelihood. Lambda varies from 0 (no influence of phylogeny) to 1 (strong phylogenetic influence under approximate Brownian motion evolution). The likelihood of resulting estimates were then compared to a likelihood value generated by assuming λ = 1 using a goodness-of-fit test with the χ2 statistic; in this case higher probability values indicate equivalence between the two models, whereas low probability values indicate a true λ<1.
(0.04 MB DOC)

Table S3 Pairwise Pearson correlation statistic between mean species trait values. Correlations which are significantly different from random (P<0.05) are in bold; correlations marginally significantly different from random (0.05<P<0.10) are in italics.
(0.04 MB DOC)

Table S4 Trait and species loadings on PCA axes developed from 9 traits of leaves from 40 species (19 exotic and 21 native to eastern North America).
(0.07 MB DOC)
