# Supplementary material for: Novel Weapons Testing: Are Invasive Plants More Chemically Defended than Native Plants?
Source: PLoS One. 2010 May 3;5(5):e10429. doi: 10.1371/journal.pone.0010429 (PMC2862706; doi:10.1371/journal.pone.0010429)
Supplement: Text S1 — Detailed methods for data collection and analysis. (0.10 MB DOC) [file pone.0010429.s001.doc]

*Study site and species*

Nearly all forests at the Smithsonian Environmental Research Center (SERC) are secondary, post-agricultural forests and range in age from ~350 years to less than 10 years since abandonment (Higman 1968, Parker et al. 2009). Typical of secondary forests in the mid-Atlantic, there are about 40 common canopy species in local forests, including tulip poplar (*Liriodendron tulipifera*), oaks (*Quercus falcata, Q. coccinea, Q. velutina, Q. rubra, Q. alba*), hickories (*Carya* spp.), beech (*Fagus grandifolia*), sweetgum (*Liquidambar styraciflora*), and red maple (*Acer rubrum*) (Higman 1968). Previous work in the area indicates that biological invasions are more prevalent in younger forests (Parker et al. 2009). The well-developed understory in these forests contains about 200 native and about 30 exotic species, including such common invaders as Japanese honeysuckle (*Lonicera japonica*), wineberry (*Rubus pheonicolasius*), and Japanese stiltgrass (*Microstegium vimineum*; Parker et al. 2009).

*Plant leaf physical traits*

Leaf toughness was measured as the grams of force required to pierce a fully expanded leaf below the inflorescence using a force gauge penetrometer (Type 516; Chatillon, Largo, Florida, USA). For each leaf we used an average of two piercings, one from each side of the mid-rib. Foliar trichome density was calculated by counting the trichomes on the tops and bottoms of leaf discs (28 mm^2^) under a dissection microscope. Specific leaf area was calculated as the area of a leaf (cm^2^ as measured with a LiCor leaf area meter) divided by its dry mass (following 24h in a drying oven at 60°C). Percentage water content was measured as ([fresh mass – dry mass] / fresh mass)*100.

*Plant leaf nutrients*

Five individual leaves from each species were randomly selected from different plants and analyzed for percentage carbon (C), nitrogen (N), phosphorus (P), and soluble protein. Percent C and N were calculated using a ~2 mg sample of finely ground plant powder in a EAI CE-440 elemental analyzer (Exeter Analytics, Coventry, UK). Percent P was determined by placing a known mass (~2 mg) of dried, ground leaf material in a muffle furnace at 550°C for two hours (Miller 1998), followed by colorimetric analysis on a microplate spectrophotometer (PowerWave XS; Biotek, Winooski, VT) using the ammonium molybdate method (Clesceri et al. 1998). Percent protein was determined by placing a known mass (~5mg) of dried plant material in 1mL NaOH for 24h at 40°C, followed by centrifuge and analysis using a modified Lowry analysis kit (Pierce, Rockford, IL) optimized for a microplate reader (PowerWave XS as above).

*Herbivore used in bioassays*

We used the woolly bear caterpillar (*Pyrrharctia isabella*) as our model herbivore. Woolly bears are abundant understory herbivores in the mid-Atlantic (E.M. Lind, *personal observation*) and individually browse a wide variety of plant species throughout their development (Wagner 2005). Individual caterpillars used in feeding trials were reared from multiple adult moths caught locally and maintained in a lab colony periodically supplemented with wild-caught moths. When not being used in feeding trials, caterpillars were fed a nutritionally complete artificial diet slightly modified from Yamamoto (1969). Diet was constructed by combining dry powders of wheat germ (32%), casein protein (20%), fructose (9%), and sucrose (9%) with Wesson salts (6.4%), cholesterol (0.3%), ascorbic acid (2.9%), sorbic acid (1%), choline (1.4%) and a vitamin mix designed for Lepidoptera (1.9%). An anti-fungal agent (FABCO-1) was included as 3.4% of the dry mass. The remainder of the diet dry mass (13%) was cellulose. The combined dry ingredients were suspended in enough 2% agar solution (m/v) to bring the diet to 83% water content. All dry ingredients were purchased from Bio-Serv (Frenchtown, NJ).

*Bioassays for activity of plant leaf chemistry*

To prepare extracts of leaf chemistry, frozen leaf tissue equivalent to 2g dry mass (based on % water content by species) was coarsely ground in a coffee grinder, added to a beaker and extracted with a series of hydrophilic to lipophilic solvents (e.g., 1:1 v/v of water:methanol, 2:1 v/v of methanol:dichloromethane, and 2:1 v/v dichloromethane:methanol). The combined extracts were then condensed by evaporating the solvents under vacuum (Savant SPD131 SpeedVac, Thermo Scientific, Waltham, MA). The resulting samples were sealed and stored at -20ºC until use in the herbivory assay.

To prepare the diet for the assays, we resuspended crude chemical extracts in acetone, and added extracts to an artificial diet comprised of 1g wheat germ, 1g cellulose powder, and 0.025g FABCO anti-fungal powder (Bio-Serv, Frenchtown, NJ). Thus, extracts from 2g dry mass of plant matter were added to 2g dry mass of artificial food. Rapid stirring in a fume hood resulted in the evaporation of the solvents but allowed the secondary extracts to adhere to the wheat germ and cellulose powder. We then added 0.75 g of agar and 10mL of boiling distilled H_2_O to the powder. This mass per volume resulted in a water content of 72.5%, equivalent to the mean (± SE) water content of all plant species that we examined (72.1 ± 1%). The resulting plant paste was stirred and immediately spread into a 5mm deep and 2cm wide mold, allowed to cool to room temperature, and the semi-solid plant gel was then removed from the mold and cut into strips prior to feeding to the herbivores. Control foods were constructed exactly the same way with the exception that the acetone did not contain plant extracts. This resulted in two diet replicates with equal nutritional content, one with the extracted chemistry of a plant species and one without.

For each plant species, we offered 20 caterpillars approximately equal sized portions of control versus test food weighed to the nearest 0.001 g. Caterpillar mass was also recorded prior to each feeding assay. Each feeding trial had a control cup containing test and control strips of food but no herbivores to control for mass loss that was unrelated to herbivory. Cups were placed into a rearing chamber set to a constant temperature of 25ºC with a 16h:8h light:dark cycle. Assay replicates were terminated when the caterpillar had eaten at least half of one diet section, or after 24h. Expected mass loss due to evaporation was calculated from the control cup as a fraction of initial mass ([mass_initial_ -mass_final_]/ mass_initial_). This correction was then applied to data from the test cup to give an expected mass lost (fraction loss_expected_ * mass_initial_). Amount eaten was then calculated as ([mass_initial_ - mass_final_] – mass_expected lost_).

*Feeding preference and exotic plant date of introduction*

We collected estimated first date of introduction for the 19 exotic plant species in the study to test whether plants more recently introduced had different chemical deterrence to a native herbivore than plants introduced long ago. The dates were collected from the Center for Invasive Species and Ecosystem Health website (<http://www.invasive.org/> accessed November 2009) supported by various sources. Five species lacked definitive dates of introduction, so we used a date in the middle of the range (e.g., “early 1600s” was coded as 1625). The dates were used in a linear regression model to predict diet preference (arcsin-square root transformed fraction eaten). Our results would have been the same if we had simply excluded species for which the date of introduction was unclear.

*Abundance of plant species at SERC*

As part of two separate studies examining plant understory communities, plant abundance was recorded over two years as percent cover by species at 68 sites spread throughout SERC. In summer of 2007, percent plant cover was estimated at each of 52 sites using the mean cover from 3 1.0 m^2^ quadrats at each site (Parker et al. 2009). In 2008, percent plant cover was estimated as the mean of 28 1.0 m^2^ quadrats from each of 16 sites (Parker et al. *unpublished data*). Individual species abundances from these surveys were pooled to generate mean per species abundance during the two years of this study. Seven species (6 exotic and 1 native) for which we had trait data were not found in the systematic surveys and were thus excluded from this analysis. The remaining 33 species were used in a factorial linear model examining the response of diet preference (arcsin-square root transformed fraction eaten) to abundance (log-transformed mean percent cover), origin (native versus exotic), and their interaction.

*Literature Cited in this Appendix*

Clesceri, L. S., A. E. Greenberg, and A. D. Eaton, editors. 1998. Standard methods for the examination of water and wastewater. American Public Health Association. , New York.

Freckleton, R. P., P. H. Harvey, and M. Pagel. 2002. Phylogenetic analysis and comparative data: A test and review of evidence. American Naturalist **160**:712-726.

Higman, D. 1968. An ecologically annotated checklist of the vascular flora at the Chesapeake Bay Center for Field Biology, with keys. Office of Ecology, Smithsonian Institution, Washington D.C.

Li, J., J. Yue, and S. Shoup. 2006. Phylogenetics of Acer (Aceroideae, Sapindaceae) based on nucleotide sequences of two chloroplast non-coding regions. Harvard Papers in Botany **11**:101-115.

Martins, E. P. and T. F. Hansen. 1997. Phylogenies and the comparative method: A general approach to incorporating phylogenetic information into the analysis of interspecific data. American Naturalist **149**:646-667

Miller, R. O. 1998. High-temperature oxidation: dry ashing.*in* Y. P. Kalra, editor. Handbook of Reference Methods for Plant Analysis. CRC Press, Boca Raton, FL.

Pagel, M. 1994. Detecting Correlated Evolution on Phylogenies - a General-Method for the Comparative-Analysis of Discrete Characters. Proceedings of the Royal Society of London Series B-Biological Sciences **255**:37-45.

Potter, D., T. Eriksson, R. C. Evans, S. Oh, J. E. E. Smedmark, D. R. Morgan, M. Kerr, K. R. Robertson, M. Arsenault, T. A. Dickinson, and C. S. Campbell. 2007. Phylogeny and classification of Rosaceae. Plant Systematics and Evolution **266**:5-43.

Team, R. D. C. 2009. R: A language and environment for statistical computing. R Foundation for Statistical Computing, Vienna, Austria.

Wagner, D. L. 2005. Caterpillars of Eastern North America: A Guide to Identification and Natural History. Princeton University Press, Princeton, NJ.

Webb, C., D. D. Ackerly, and S. W. Kembel. 2008. Phylocom: software for the analysis of phylogenetic community structure and trait evolution. Bioinformatics **24**:2098-2100.

Yamamoto, R. T. 1969. MASS REARING OF TOBACCO HORNWORM .2. LARVAL REARING AND PUPATION. Journal of Economic Entomology **62**:1427-&.
