# Supplementary material for: Novel Weapons Testing: Are Invasive Plants More Chemically Defended than Native Plants?
Source: PLoS One. 2010 May 3;5(5):e10429. doi: 10.1371/journal.pone.0010429 (PMC2862706; doi:10.1371/journal.pone.0010429)
Supplement: Table S1 — Taxonomic, origin, and trait data for species used in the study. Data are species means based on the indicated replications per species. Diet preference represents the mean fraction consumed of artificial diet with extracts from each species versus a neutral control diet (0 = all control preferred, 1 = all species extract preferred). Origin and date introduced for exotic species compiled from www.invasive.org. “NA” = North America (native species). (0.10 MB DOC) [file pone.0010429.s002.doc]

| **Family** | **Species** | **native region (Date Introduced)** | **Date leaves collected** | **n = 20 individuals / sp** | | | | **n= 5 individuals / sp** | | | | **Diet preference** |
| --- | --- | --- | --- | --- | --- | --- | --- | --- | --- | --- | --- | --- |
|  |  |  |  | **% water** | **SLA (cm^2^/g)** | **leaf toughness (N)** | **trichomes (cm^-2^)** | **%C** | **%N** | **%P** | **% soluble Protein** |  |
| Anacardiaceae | *Toxicodendron radicans* (L.) Kuntze | NA | 20-Jul-09 | 71.61 | 427.55 | 1.15 | 135.70 | 43.15 | 2.32 | 0.35 | 0.12 | 0.55 |
| Annonaceae | *Asimina triloba* (L.) Dunal | NA | 22-Jul-08 | 75.72 | 416.86 | 1.29 | 70.50 | 47.12 | 3.15 | 0.22 | 0.07 | 0.19 |
| Araceae | *Arisaema triphyllum* (L.) Schott | NA | 8-Jul-08 | 85.46 | 514.16 | 1.41 | 0.10 | 44.02 | 2.84 | 0.21 | 0.09 | 0.54 |
| Araliaceae | *Hedera helix* L. | Europe (1600s) | 6-Jul-09 | 70.75 | 191.11 | 2.64 | 0.00 | 44.92 | 1.99 | 0.25 | 0.08 | 0.21 |
| Asteraceae | *Cirsium arvense* (L.) Scop. | Eurasia (1600s) | 2-Jul-09 | 83.55 | 213.46 | 1.16 | 1586.55 | 43.42 | 2.81 | 0.23 | 0.11 | 0.55 |
| Asteraceae | *Eupatorium purpureum* L. | NA | 1-Jul-09 | 78.36 | 258.64 | 0.73 | 408.55 | 46.39 | 3.45 | 0.35 | 0.15 | 0.68 |
| Asteraceae | *Verbesina alternifolia* (L.) Britton ex Kearney | NA | 13-Jul-09 | 72.11 | 294.77 | 0.58 | 330.10 | 42.97 | 3.68 | 0.45 | 0.17 | 0.50 |
| Berberidaceae | *Berberis thunbergii* DC. | Asia (1864) | 5-Sep-08 | 68.87 | 222.17 | 1.70 | 0.65 | 44.56 | 1.56 | 0.53 | 0.17 | 0.32 |
| Betulaceae | *Carpinus caroliniana* Walter | NA | 10-Sep-08 | 56.43 | 318.21 | 1.15 | 34.35 | 47.51 | 2.18 | 0.18 | 0.12 | 0.47 |
| Brassicaceae | *Alliaria petiolata* (M. Bieb.) Cavara & Grande | Europe (1800s) | 2-Jul-09 | 86.29 | 606.51 | 0.31 | 0.00 | 36.78 | 2.91 | 0.72 | 0.12 | 0.20 |
| Caprifoliaceae | *Lonicera japonica* Thunb. | Asia (1806) | 9-Jul-08 | 74.66 | 374.94 | 1.12 | 15.00 | 42.73 | 1.75 | 0.34 | 0.10 | 0.60 |
| Caprifoliaceae | *Viburnum prunifolium* L. | NA | 6-Jul-09 | 72.93 | 268.75 | 1.06 | 0.00 | 43.55 | 1.70 | 0.20 | 0.07 | 0.01 |
| Celastraceae | *Celastrus orbiculatus* Thunb. | China (1860) | 1-Jul-09 | 80.44 | 300.45 | 0.79 | 0.20 | 43.28 | 2.94 | 0.25 | 0.19 | 0.12 |
| Cornaceae | *Cornus florida* L. | NA | 11-Sep-08 | 64.56 | 379.96 | 1.65 | 433.85 | 44.59 | 1.52 | 0.13 | 0.08 | 0.20 |
| Elaeagnaceae | *Elaeagnus umbellate* Thunb. | China & Japan (1830) | 2-Sep-08 | 70.35 | 253.65 | 1.08 | 465.40 | 48.10 | 3.59 | 0.16 | 0.14 | 0.75 |
| Fabaceae | *Albizia julibrissin* Durazz. | Asia (1745) | 18-Sep-08 | 63.68 | 209.74 | 1.28 | 80.25 | 47.50 | 3.79 | 0.23 | 0.15 | 0.11 |
| Fabaceae | *Pueraria montana* (Lour.) Merr. | Asia (1876) | 28-Aug-08 | 68.50 | 320.30 | 1.62 | 895.40 | 46.66 | 4.85 | 0.31 | 0.13 | 0.86 |
| Fagaceae | *Fagus grandifolia* Ehrh. | NA | 25-Aug-08 | 50.77 | 346.79 | 1.46 | 118.80 | 47.66 | 2.17 | 0.13 | 0.11 | 0.65 |
| Hamamelidaceae | *Liquidambar styraciflua* L. | NA | 17-Jul-08 | 72.94 | 300.93 | 1.58 | 1.65 | 47.24 | 1.73 | 0.26 | 0.10 | 0.67 |
| Lauraceae | *Lindera benzoin* (L.) Blume | NA | 3-Jul-08 | 75.52 | 403.22 | 1.47 | 13.70 | 47.89 | 3.28 | 0.27 | 0.10 | 0.28 |
| Magnoliaceae | *Liriodendron tulipifera* L. | NA | 18-Jul-08 | 77.72 | 382.92 | 1.55 | 15.80 | 46.87 | 2.20 | 0.15 | 0.11 | 0.22 |
| Oleaceae | *Ligustrum sinense* Lour. | China (1852) | 13-Jul-09 | 71.50 | 288.64 | 1.43 | 0.35 | 46.85 | 1.98 | 0.14 | 0.10 | 0.48 |
| Onagraceae | *Circaea lutetiana* L. | NA | 11-Jul-08 | 86.16 | 709.80 | 1.13 | 4.05 | 42.57 | 2.61 | 0.23 | 0.11 | 0.47 |
| Platanaceae | *Platanus occidentalis* L. | NA | 16-Sep-08 | 65.20 | 246.60 | 1.26 | 0.00 | 48.98 | 2.53 | 0.21 | 0.19 | 0.69 |
| Poaceae | *Cinna arundinacea* L. | NA | 17-Jul-09 | 77.82 | 565.71 | 1.17 | 0.40 | 42.25 | 3.34 | 0.33 | 0.14 | 0.33 |
| Poaceae | *Microstegium vimineum* (Trin.) A. Camus | Asia (1920) | 16-Jul-08 | 56.56 | 722.26 | 1.17 | 112.20 | 45.02 | 2.97 | 0.32 | 0.17 | 0.67 |
| Polygonaceae | *Persicaria perfoliata* (L.) H. Gross | east Asia (1937) | 11-Jul-08 | 85.65 | 906.32 | 0.81 | 1.30 | 43.94 | 3.39 | 0.29 | 0.09 | 0.34 |
| Polygonaceae | *Polygonum cuspidatum* Siebold & Zucc. | east Asia (late 1800s) | 15-Sep-08 | 74.23 | 244.23 | 1.76 | 11.05 | 47.11 | 2.65 | 0.22 | 0.19 | 0.60 |
| Polygonaceae | *Polygonum virginianum* L. | NA | 14-Jul-08 | 81.38 | 417.15 | 2.12 | 80.45 | 42.79 | 2.79 | 0.21 | 0.09 | 0.62 |
| Rosaceae | *Duchesnea indica* (Andrews) Focke | Asia (1890) | 10-Jul-08 | 72.00 | 295.98 | 1.57 | 85.55 | 42.64 | 1.83 | 0.38 | 0.12 | 0.53 |
| Rosaceae | *Rosa multiflora* Thunb. | Asia (1866) | 7-Jul-08 | 62.73 | 417.10 | 1.00 | 133.60 | 44.04 | 2.08 | 0.17 | 0.13 | 0.61 |
| Rosaceae | *Rubus occidentalis* L. | NA | 1-Jul-09 | 65.66 | 228.32 | 0.66 | 290.60 | 46.56 | 2.32 | 0.20 | 0.16 | 0.44 |
| Rosaceae | *Rubus phoenicolasius* Maxim. | east Asia (1890) | 7-Jul-08 | 69.75 | 443.18 | 1.30 | 29651.15 | 45.89 | 2.74 | 0.22 | 0.08 | 0.83 |
| Sapindaceae | *Acer negundo* L. | NA | 22-Jul-08 | 77.40 | 376.62 | 1.01 | 9.15 | 44.67 | 2.56 | 0.24 | 0.13 | 0.50 |
| Sapindaceae | *Acer platanoides* L. | Europe (1756) | 23-Jul-08 | 59.09 | 207.59 | 1.49 | 0.15 | 45.92 | 2.52 | 0.62 | 0.14 | 0.64 |
| Sapindaceae | *Acer rubrum* L. | NA | 21-Jul-08 | 59.42 | 261.31 | 1.36 | 0.70 | 48.30 | 1.88 | 0.15 | 0.21 | 0.49 |
| Scrophulariaceae | *Paulownia tomentosa* (Thunb.) Siebold & Zucc. ex Steud. | east Asia (early 1800s) | 3-Sep-08 | 74.32 | 197.26 | 1.13 | 456.05 | 47.79 | 2.04 | 0.20 | 0.20 | 0.14 |
| Simaroubaceae | *Ailanthus altissima* (Mill.) Swingle | Asia (1748) | 14-Jul-08 | 76.92 | 391.59 | 1.16 | 57.15 | 45.07 | 3.93 | 0.32 | 0.11 | 0.46 |
| Smilacaceae | *Smilax rotundifolia* L. | NA | 10-Jul-08 | 66.48 | 231.39 | 2.01 | 0.00 | 47.29 | 1.81 | 0.11 | 0.09 | 0.65 |
| Vitaceae | *Parthenocissus quinquefolia* (L.) Planch. | NA | 9-Jul-08 | 79.96 | 416.21 | 1.77 | 12.80 | 44.12 | 2.15 | 0.21 | 0.07 | 0.60 |
