# Supplementary material for: Novel Weapons Testing: Are Invasive Plants More Chemically Defended than Native Plants?
Source: PLoS One. 2010 May 3;5(5):e10429. doi: 10.1371/journal.pone.0010429 (PMC2862706; doi:10.1371/journal.pone.0010429)
Supplement: Table S2 — Estimates of Pagel's λ showing influence of assembled phylogeny on trait variance across species. For each trait individually, species mean values and the assembled phylogeny (see supplemental methods) were used to fit a λ value with restricted maximum likelihood. Lambda varies from 0 (no influence of phylogeny) to 1 (strong phylogenetic influence under approximate Brownian motion evolution). The likelihood of resulting estimates were then compared to a likelihood value generated by assuming λ = 1 using a goodness-of-fit test with the χ2 statistic; in this case higher probability values indicate equivalence between the two models, whereas low probability values indicate a true λ<1. (0.04 MB DOC) [file pone.0010429.s003.doc]

| **trait** | **λ** | **LnL(lambda)** | **LnL(Brownian)** | **p(χ^2^)** |
| --- | --- | --- | --- | --- |
| Leaf area | 1.00E-07 | -214.01 | -228.14 | <0.0001 |
| % water | 6.40E-07 | -147.88 | -152.68 | 0.002 |
| SLA | 1.00E-07 | -1353.45 | -1964.73 | <0.0001 |
| toughness | 0.067904 | -248.64 | -284.91 | <0.0001 |
| trichomes | 0.604245 | -97.28 | -98.30 | **0.154** |
| %C | 1.56E-05 | -95.87 | -98.60 | 0.0193 |
| %N | 6.56E-07 | -46.59 | -48.94 | 0.030 |
| %P | 9.44E-06 | 20.63 | 18.66 | 0.047 |
| %Protein | 9.70E-06 | 69.96 | 67.91 | 0.043 |
| caterpillar feeding preference | 1.70E-05 | 2.80 | -1.17 | 0.005 |
