# Supplementary material for: Novel Weapons Testing: Are Invasive Plants More Chemically Defended than Native Plants?
Source: PLoS One. 2010 May 3;5(5):e10429. doi: 10.1371/journal.pone.0010429 (PMC2862706; doi:10.1371/journal.pone.0010429)
Supplement: Table S3 — Pairwise Pearson correlation statistic between mean species trait values. Correlations which are significantly different from random (P<0.05) are in bold; correlations marginally significantly different from random (0.05<P<0.10) are in italics. (0.04 MB DOC) [file pone.0010429.s004.doc]

|  | % water | SLA | toughness | trichomes | %C | %N | %P | % protein |
| --- | --- | --- | --- | --- | --- | --- | --- | --- |
| % water |  |  |  |  |  |  |  |  |
| SLA | **0.37** |  |  |  |  |  |  |  |
| toughness | -0.18 | -0.29 |  |  |  |  |  |  |
| trichomes | -0.04 | 0.07 | -0.01 |  |  |  |  |  |
| %C | **-0.54** | **-0.47** | *0.30* | 0.05 |  |  |  |  |
| %N | 0.23 | 0.23 | *-0.31* | 0.05 | 0.00 |  |  |  |
| %P | 0.17 | 0.14 | *-0.31* | -0.06 | **-0.57** | 0.20 |  |  |
| % protein | *-0.27* | *-0.27* | **-0.35** | -0.17 | 0.21 | 0.15 | 0.20 |  |
| deterrent chemistry | 0.23 | 0.00 | -0.12 | **-0.29** | -0.21 | -0.16 | 0.04 | -0.06 |
