# Supplementary material for: Novel Weapons Testing: Are Invasive Plants More Chemically Defended than Native Plants?
Source: PLoS One. 2010 May 3;5(5):e10429. doi: 10.1371/journal.pone.0010429 (PMC2862706; doi:10.1371/journal.pone.0010429)
Supplement: Table S4 — Trait and species loadings on PCA axes developed from 9 traits of leaves from 40 species (19 exotic and 21 native to eastern North America). (0.07 MB DOC) [file pone.0010429.s005.doc]

| TRAIT | PC1 | PC2 | PC3 | PC4 | PC5 | PC6 | PC7 | PC8 | PC9 |
| --- | --- | --- | --- | --- | --- | --- | --- | --- | --- |
| %H_2_O | 1.034 | 0.270 | -0.330 | 0.345 | -0.419 | 0.342 | -0.617 | 0.021 | 0.195 |
| SLA | 0.956 | 0.011 | -0.568 | -0.006 | 0.107 | -0.830 | 0.203 | 0.280 | 0.158 |
| toughness | -0.737 | 0.817 | -0.360 | -0.431 | -0.499 | 0.263 | 0.172 | 0.455 | 0.015 |
| trichomes | -0.312 | -0.870 | -0.686 | 0.153 | 0.598 | 0.535 | 0.045 | 0.261 | 0.141 |
| %C | -1.227 | -0.213 | -0.029 | 0.368 | -0.359 | -0.245 | 0.108 | -0.209 | 0.386 |
| %N | 0.427 | -0.969 | -0.399 | 0.279 | -0.728 | 0.118 | 0.371 | -0.035 | -0.200 |
| %P | 0.871 | -0.320 | 0.595 | -0.749 | -0.092 | 0.329 | 0.325 | -0.084 | 0.277 |
| protein | -0.245 | -0.806 | 0.994 | 0.080 | -0.177 | -0.212 | -0.321 | 0.445 | -0.005 |
| deterrent.chem | 0.431 | 0.635 | 0.615 | 0.891 | 0.134 | 0.257 | 0.457 | 0.156 | 0.045 |

| SPECIES | PC1 | PC2 |
| --- | --- | --- |
| *Albizia julibrissin* | -0.413 | -0.475 |
| *Elaeagnus umbellata* | -0.603 | -1.059 |
| *Berberis thunbergii* | -0.008 | 0.499 |
| *Fagus grandifolia* | -1.097 | -0.136 |
| *Celastrus orbiculatus* | 0.604 | -0.044 |
| *Acer negundo* | 0.244 | -0.049 |
| *Cirsium arvense* | 0.163 | -0.359 |
| *Ligustrum sinense* | -0.427 | 0.736 |
| *Cinna arundinacea* | 0.939 | 0.012 |
| *Cornus florida* | -0.396 | 0.837 |
| *Circaea lutetiana* | 1.03 | 0.377 |
| *Hedera helix* | -0.497 | 1.681 |
| *Eupatorium purpureum* | 0.112 | -1.198 |
| *Alliaria petiolata* | 2.505 | -0.074 |
| *Smilax rotundifolia* | -0.954 | 0.984 |
| *Lonicera japonica* | 0.363 | 0.313 |
| *Duchesnea indica* | 0.091 | 0.266 |
| *Carpinus caroliniana* | -0.732 | -0.047 |
| *Arisaema triphyllum* | 0.575 | 0.556 |
| *Microstegium vimineum* | 0.072 | -0.893 |
| *Polygonum cuspidatum* | -0.578 | -0.164 |
| *Pueraria montana* | -0.319 | -1.407 |
| *Persicaria perfoliata* | 1.458 | 0.128 |
| *Rosa multiflora* | -0.216 | -0.253 |
| *Acer platanoides* | -0.179 | -0.217 |
| *Asimina triloba* | 0.105 | 0.244 |
| *Toxicodendron radicans* | 0.323 | -0.178 |
| *Paulownia tomentosa* | -0.512 | -0.329 |
| *Acer rubrum* | -0.999 | -0.105 |
| *Rubus occidentalis* | -0.432 | -0.7 |
| *Lindera benzoin* | 0.001 | 0.173 |
| *Liquidambar styraciflua* | -0.445 | 0.603 |
| *Platanus occidentalis* | -0.811 | -0.355 |
| *Polygonum virginicanum* | 0.144 | 0.511 |
| *Ailanthus altissima* | 0.398 | -0.537 |
| *Liriodendron tulipifera* | -0.165 | 0.689 |
| *Verbesina alternifolia* | 0.588 | -1.359 |
| *Viburnum prunifolium* | 0.284 | 1.272 |
| *Parthenocissus quinquefolia* | 0.109 | 0.82 |
| *Rubus phoenicolasius* | -0.321 | -0.76 |
